# Support Vector Machine-Based Transmit Antenna Allocation for Multiuser Communication Systems

**DOI:** 10.3390/e21050471

**Published:** 2019-05-06

**Authors:** Huifa Lin, Won-Yong Shin, Jingon Joung

**Affiliations:** 1 Telecommunication and Image Technology Laboratories, Sharp Corporation, Chiba 261-8520, Japan; 2 Computational Science and Engineering, Yonsei University, Seoul 03722, Korea; 3 School of Electrical and Electronics Engineering, Chung-Ang University, Seoul 06974, Korea

**Keywords:** antenna allocation systems, multiuser communication systems, multiclass classification, supervised machine learning, support vector machine

## Abstract

In this paper, a support vector machine (SVM) technique has been applied to an antenna allocation system with multiple antennas in multiuser downlink communications. Here, only the channel magnitude information is available at the transmitter. Thus, a subset of transmit antennas that can reduce multiuser interference is selected based on such partial channel state information to support multiple users. For training, we generate the feature vectors by fully utilizing the characteristics of the interference-limited setup in the multiuser downlink system and determine the corresponding class label by evaluating a key performance indicator, i.e., sum rate in multiuser communications. Using test channels, we evaluate the performance of our antenna allocation system invoking the SVM-based allocation and optimization-based allocation, in terms of sum-rate performance and computational complexity. Rigorous testing allowed for a comparison of a SVM algorithm design between one-vs-one (OVO) and one-vs-all (OVA) strategies and a kernel function: (i) OVA is preferable to OVO since OVA can achieve almost the same sum rate as OVO with significantly reduced computational complexity, (ii) a Gaussian function is a good choice as the kernel function for the SVM, and (iii) the variance (kernel scale) and penalty parameter (box constraint) of an SVM kernel function are determined by 21.56 and 7.67, respectively. Further simulation results revealed that the designed SVM-based approach can remarkably reduce the time complexity compared to a traditional optimization-based approach, at the cost of marginal sum rate degradation. Our proposed framework offers some important insights for intelligently combining machine learning techniques and multiuser wireless communications.

## 1. Introduction

Recently, machine learning has been attracting much research interest from various fields due to numerous successful applications to solve significant practical problems [1,2,3,4,5,6,7,8,9,10,11]. Most of the conventional approaches in communication system design rely on maximizing or minimizing the objective functions, i.e., optimization-driven approaches. However, for some problems, one has to resort to algorithms with fast-increasing complexity, e.g., the antenna selection/allocation problem in multiuser communication systems [12,13,14,15]. Hence, for future application scenarios with large-scale configurations, such as massive multiple-input and multiple-output (MIMO) systems and machine learning-based methods, a data-driven approach seems to be more promising because it is possible to provide near-optimal communication performance with relatively low online prediction complexity, leaving the high complexity part to the offline training phase in machine learning. In the research fields of communication and signal processing, different types of machine learning methods including (i) unsupervised learning, (ii) reinforcement learning, and (iii) supervised learning find suitable application areas in many areas [16,17,18,19]. In this study, we attempt to explore the possibility of applying supervised learning techniques (switching the optimization-driven thinking to the data-driven thinking) to solve a specific antenna allocation problem in multiuser communication systems. Even compared to the optimization-driven antenna selection algorithms with reduced complexity [13,20,21,22,23,24,25], our proposed supervised learning-based approach is a promising alternative since it demonstrates almost optimal performance while greatly reducing complexity. Moreover, our supervised learning-based approach can also provide design flexibility based on a trade-off between computational complexity and sum-rate performance by adjusting the number of effective classes, i.e., computational complexity can be further reduced by using a smaller number of effective classes, with tolerable sum-rate performance degradation.

### 1.1. Related Work

An early study investigated dynamic channel assignment for cellular systems by using neural networks [26]. One of the most popular unsupervised learning methods, *k*-means clustering, was used to jointly optimize both the gateway partitioning and the virtual-channel allocation in hybrid optical/wireless networks [27], to group distributed users in networks [28], and to blindly detect symbols [29]. Decision trees and neural networks were applied to enhance the routing performance [30].

Reinforcement learning techniques have also been found to be helpful in solving multi-armed bandit problems in wireless communications [31,32], link adaptation [33], and to autonomously manage heterogeneous networks [34]. Other reinforcement learning methods, such as Markov decision processes and Q-learning, have been applied to energy harvesting sensor networks [35], cognitive radio networks [36], and opportunistic access of macro/femtocell heterogeneous networks [37]. Accelerated reinforcement learning algorithms have been used to predict spectrum opportunities in opportunistic spectrum access networks, resulting in improved secondary sum-rate performance [38].

Supervised learning methods, such as the *k*-nearest neighbors algorithm (*k*-NN) and the support vector machine (SVM), have been employed to design link adaptation methods for wireless communication networks [39,40,41]. There have been extensive studies on not only improving the accuracy or complexity of SVMs [42], but also applying SVM to various classification fields such as signal detection in visible light communications [43]. The efficiency of *k*-NN-based beam allocation [44] was also demonstrated in multiuser massive MIMO systems. The whole communication system was interpreted as a machine learning system, i.e., mapping major components in the communication system including the transmitters, channels, and receivers to different layers in a neural network [45,46]. In [47], the communication system was considered as an autoencoder and the optimization of the communication system was accomplished by training the corresponding neural network.

Among the attempts to apply machine learning to the communication and signal processing fields, and specifically to the antenna allocation problem considered here, a recent work has applied supervised machine learning-based methods for antenna allocation in multi-antenna point-to-point (single-user) wireless networks [48]. It was shown that the antenna allocation in wireless communications can be converted to a multiclass classification problem. Compared to the conventional optimization-driven methods in [12,13,14], the multiclass classification, learning-based method in [48] provided near-optimal communication performance with relatively low online computational complexity.

It is worth noting that computational complexity is one of the bottlenecks for traditional optimization-driven antenna selection approaches because of the rapidly increasing complexity of exhaustive search algorithms for the larger number of antennas [13,20]. To reduce computational complexity, seminal studies have proposed optimization-driven antenna selection methods in single-user communication systems [21,22]. For multiuser communication systems, there have also been many studies attempting to reduce the complexity of antenna selection [23,24,25].

### 1.2. Motivations and Contributions

Inspired by the pioneering work on learning-based antenna allocation for a single-user system [48], we focus on a wireless downlink multiuser communication system with antenna allocation. Extending the single-user system to multiuser networks, it is important to properly determine one or more communication performance metrics, such as sum rate and bit error rate (BER) [49]. For multiuser communication systems, maximizing the system sum rate might be a proper design target rather than minimizing the BER, due to the presence of interference among multiple users. Moreover, it is easy to obtain a rather trivial solution to the BER minimization problem for a multiuser communication system by selecting only one user (but not multiple users). In [41], learning-based link adaptation methods were designed for multiuser communication networks, where a greedy algorithm was employed for user selection. However, rather than improving fairness by user scheduling, our aim is to maximize the sum rate of all the users in the network by allocating the proper antenna to each user. Hence, we first formulate antenna allocation in the multiuser network as a multiuser sum rate maximization problem and solve it by using multiclass classification algorithms. To the best of our knowledge, this study is the first attempt at machine learning-based antenna allocation for multiuser wireless networks.

**Figure 1:**
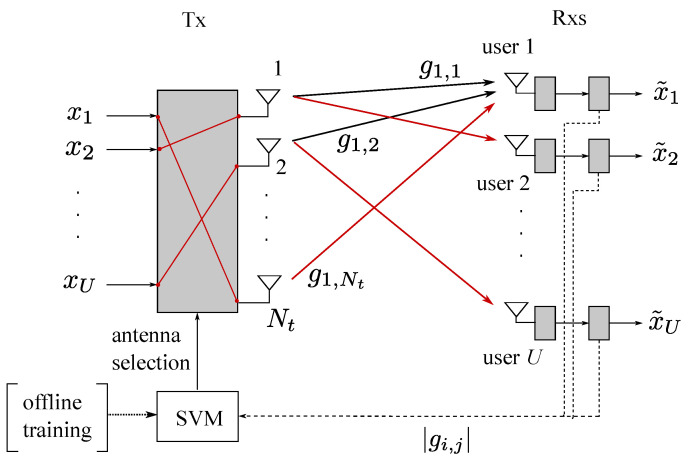
System model of the considered multiuser communication network consisting of one transmitter with $N_{t}$ antennas and *U* users with a single antenna.

The main contributions of this study are summarized as follows:

- We interpret the antenna allocation system for multiuser communication as a multiclass classification learning system. For the components of the learning system, such as the training data and the corresponding class labels, we first model the counterparts in the conventional communication system, and we then construct them with a proper format for the learning system.
- We establish a communication system with an SVM module that allocates transmit antennas to each user in multiuser communication networks with partial channel state information at the transmitter (CSIT), as shown in Figure 1. The antenna allocation method is designed for a frequency-division duplex (FDD) system, where only quantized channel gain information is available.
- The parameters of the designed SVM are tuned based on extensive numerical experiments in order to improve the sum-rate performance of the communication system. For the designed SVM, we find that a Gaussian function is a good choice for the kernel function, which is one of the most important parameters for tuning SVMs. (Artificial neural networks (ANNs) can also be employed in our learning system, which may result in a slightly better sum-rate performance at the expense of both higher computational complexity and a larger training dataset than for *k*-NN and SVM. Thus, ANNs are not considered in this study as our main focus is on the design of learning systems showing a significant reduction in complexity from the optimization-based approach with marginal sum rate reduction.) From our rigorous simulation, the variance (kernel scale) and penalty parameter (box constraint) of an SVM kernel function are determined by 21.56 and 7.67, respectively.
- Numerical experiments are extensively performed for various configurations of communication systems in order to evaluate the proposed SVM-based antenna allocation method. We find that with lower online computational complexity, the designed SVM method achieves near-optimal performance, as is obtained from the conventional optimization approach. Compared to the *k*-NN method, the SVM method is superior not only in terms of sum-rate performance but also prediction complexity performance.

### 1.3. Organization

The rest of the paper is organized as follows. In Section 2, the system model, optimization problem formulation, and corresponding optimization-driven solutions are described. In Section 3, we introduce the proposed machine learning-based antenna allocation method from overall framework structure to implementation details. In Section 4, the proposed method is evaluated to determine the parameters of the designed learning system, and its performance is compared to that of the conventional optimization-driven method. Finally, we conclude this paper in Section 5.

## 2. System Model and Conventional Optimization Approach

### 2.1. System Model

As illustrated in Figure 1, we consider a multiuser communication network consisting of one multi-antenna transmitter (Tx) and *U* selected/scheduled single-antenna receivers (Rxs or users). Let $N_{t}\geq U$ denote the number of antennas at the Tx. We use $h_{i}\in C^{N_{t}\times 1}$ to denote the channel coefficient vector from the transmitter to receiver *i*, where i∈{1,2,⋯,U}. Let $H\in C^{U\times N_{t}}$ be an overall channel coefficient matrix from the transmitter to all the receivers, where the *i*th row of H is given by $h_{i}^{T}$ and the (i,j)th entry of H is expressed as $h_{i,j}$, that is, the channel from Tx antenna *j* to user *i*. In this study, we consider a low-cost Tx that has a limited number of radio frequency (RF) chains and computational capability. Thus, an antenna selection scheme using a part of transmit antennas is relevant, rather than the highly complex optimal multiuser pre-processing or beamforming schemes. Accordingly, channel magnitude information, i.e., partial CSI, is sufficient for simple antenna selection at the Tx (refer to Section 3 for the overall procedure). Note that the main motivation of this study is to effectively reduce the computational complexity at the Tx by using a machine learning-based method. The partial CSIT is available through channel gain feedback from Rxs to Tx. Specifically, each Rx *i* estimates its own channel gains, i.e., $h_{i,j}$ for all *j*, obtains their magnitude information $g_{i,j}={\left|h_{i,j}\right|}^{2}$, and feeds them back to the Tx. The Tx accumulates the feedback information from all Rxs and constructs a channel gain matrix $G\in R^{U\times N_{t}}$ whose real-value entries are given by $g_{i,j}={\left|h_{i,j}\right|}^{2}$.

### 2.2. Optimization Problem Formulation

The Tx selects *U* antennas from $N_{t}$ antennas to allocate one antenna to each user. Then, *U* independent data streams are transferred to the *U* users, i.e., data stream $x_{i}$ is delivered to user *i* and is estimated by ${\tilde{x}}_{i}$. For a certain antenna allocation scheme indexed by *l*, let $s_{i}^{\left(l\right)}$ denote the index of the antenna allocated to user *i*, where $s_{i}^{\left(l\right)}\in \left\{1,2,\cdots ,N_{t}\right\}$ and $s_{i}^{\left(l\right)}\neq s_{j}^{\left(l\right)}$ if i≠j (i,j∈{1,2,⋯,U}). The corresponding index vector of the *U* allocated antennas is then defined as $s_{l}≜{\left[s_{1}^{\left(l\right)},s_{2}^{\left(l\right)},\cdots ,s_{U}^{\left(l\right)}\right]}^{T}$, where the superscript *T* represents the transpose of a vector or matrix, and index l∈L is used to denote the antenna allocation scheme. Here, we define L≜{1,2,⋯,L} as a set of antenna allocation schemes, where the number of all available $s_{l}$ (all valid antenna allocation schemes), *L*, is given by

(1) $$L≜\frac{N_{t}!}{(N_{t}-U)!}.$$

Now, suppose that the antenna allocation scheme is given by $s_{l}$. We can then compute the resultant data rate of user *i* as follows [50]:

(2) Ri(sl)=log21+PTXgi,si(l)N0+∑j=1j≠iUPTXgi,sj(l),

where $P_{\mathrm{TX}}$ is the transmit power, $N_{0}$ is the noise variance, $P_{\mathrm{TX}}g_{i,s_{i}^{\left(l\right)}}$ is the power of the desired signal to user *i*, and ∑j=1j≠iUPTXgi,sj(l) is the power of interference signals from the antennas allocated to other users. Then, the sum rate of the system for the antenna allocation $s_{l}$ is given by

(3) $$R_{\mathrm{sum}}\left(s_{l}\right)=\sum_{i=1}^{U}R_{i}\left(s_{l}\right).$$

The optimization problem is then formulated as follows:

(4) $$\begin{array}{cc} \underset{l\in L}{\mathrm{maximize}} & R_{\mathrm{sum}}\left(s_{l}\right). \end{array}$$

### 2.3. Optimization-Driven Solutions

To solve the formulated combinatorial optimization problem in Equation (4), a brute-force exhaustive search (or any other more sophisticated optimization-driven algorithms) with high computational complexity can be applied to find $l^{*}$ that maximizes $R_{\mathrm{sum}}$. First, the data rates of *U* users, i.e., $R_{i}\left(s_{l}\right),i\in \left\{1,\cdots ,U\right\}$, are computed by using Equation (2) for all the *L* antenna allocation schemes. Then, the sum rate can be computed based on (3). Among the antenna allocation schemes, the optimal index $l^{*}$ that maximizes the value of $R_{\mathrm{sum}}$ is determined. For each antenna allocation scheme, the exhaustive search algorithm traverses all *U* users to compute the data rate, where (U−1) computations are required to calculate the total interference term. Thus, the computational complexity of this exhaustive search is given by $O\left(LU\left(U-1\right)\right)=O\left(\frac{U^{2}N_{t}!}{(N_{t}-U)!}\right)$. It can be seen that the complexity increases and becomed prohibitively large as $N_{t}$ or *U* increases.

For better readability, the main notations used here to describe communication systems are summarized in Table 1.

## 3. SVM-Based Antenna Allocation

In order to reduce the computational complexity of the exhaustive search for antenna selection, we consider SVM-based antenna selection to solve Equation (4). Specifically, we employ a multiclass SVM algorithm to classify channel gain samples into *L* classes, each of which corresponds to an available antenna allocation scheme. With a sufficient number of channel gain samples, i.e., training data, we can design a classification model, which can be used to predict the class of a test channel gain matrix, i.e., the best antenna allocation scheme for a new channel realization in a test, i.e., actual communications.

**Figure 2:**
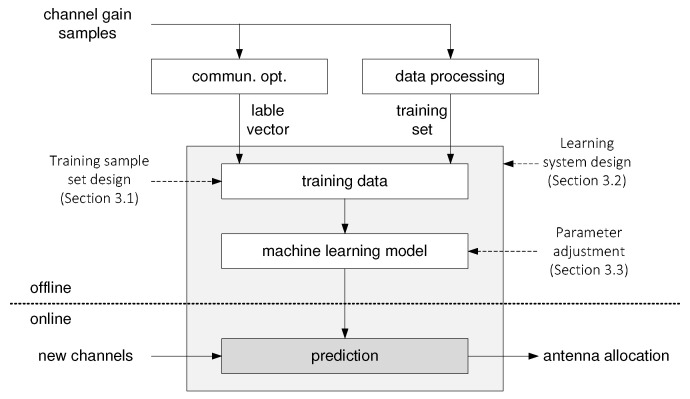
Machine learning framework for antenna allocation in a multiuser communication system.

Generally speaking, in a machine learning system, a learning model is first trained by the input training set and the corresponding labels [51,52]. Then, this learning model can be used to predict the class labels for a new test dataset. The overall machine learning framework of our antenna allocation system is illustrated in Figure 2. It is worth noting that there are two types of tasks in this framework, an offline task and an online task. The online task includes channel estimation and channel allocation through learning-based prediction. The offline task consists of three tasks: i) training sample set design, ii) learning systems design, and iii) parameter adjustment. Distinguishing the online and offline tasks is crucial in communication systems. This is because the offline task can be performed with more powerful computing resources and relaxed computational complexity requirements, while the online task typically has stringent latency and computing constraints. In the following three subsections, the three offline tasks will be described in detail.

### 3.1. Task 1: Designing a Training Sample Set

We need to manipulate the matrix form of the channel gain samples into training data with a suitable form for input into the learning system. Three procedures are performed to obtain the training data for the machine learning system (not necessarily in sequence): (i) design training data from the channel gain matrices, (ii) design the key performance indicator (KPI), and (iii) declare the corresponding label based on the KPI, i.e., labeling.

#### 3.1.1. Subtask 1-1: KPI Design

A KPI is designed to label the training set. In general, a KPI can be defined as any metric used in communications, such as spectral efficiency, energy efficiency, BER, effective signal-to-noise ratio (SNR), communications latency, and any combination thereof [53]. In this study, we use the sum rate of a system, $R_{\mathrm{sum}}$, as the target KPI.

#### 3.1.2. Subtask 1-2. Training Set Design

The training samples are the input for a learning system and are known as input variables, predictors, or attributes. As shown in Section 2.1, we assume that magnitude channel information, i.e., the channel gain matrix G, is available in our communication system. Based on the available channel information, it is important to properly design the training set by taking into account not only the target KPI, which affects communication performance, but also the complexity of the system. For example (refer to [54] and the references therein), singular values are used for a singular value-based antenna selection system, the minimum eigenvalues of the Hermitian matrix of the channel matrix for Gerschgorin circle-based antenna selection systems, channel norm values for norm-based antenna selection systems, and the dot products of channel column vectors for correlation-based antenna selection systems. Here, the singular values are clearly a good candidate for a training set for various KPIs (e.g., spectral efficiency, energy efficiency, and BER), yet they require a higher complexity higher than the other values. In this study, we adopt a signal-to-interference-leakage ratio (SILR) metric that is closely coupled with the sum rate, which is our target KPI. It is worth noting that with this SILR metric, it is sufficient to perform antenna allocation with the channel gain knowledge at the Tx, even without knowing the signal-to-interference-plus-noise ratio (SINR) of the users. We also note that it may not be possible to acquire the received SINR at the Tx under our communication mechanism using the channel gain feedback. This is because the sum of interference links can be computed after the antenna allocation process and the set of allocated antennas is not available in the training phase.

An SILR matrix, $Z\in R^{U\times N_{t}}$, is employed to generate the training set, which can be computed based on G. Specifically, the entry of the *i*th row and the *j*th column of Z is given by

(5) Zi,j=gi,j∑k=1k≠jUgi,k.

From Equation (5), it can be seen that the SILR metric simultaneously captures both the desired signal strength and the interference leakage to other users. Because a machine learning system requires the real-value vector input of multiple features, we transform the SILR matrix into a $R^{1\times N_{t}U}$ vector by stacking *U* users’ vectors (the row vectors in Z). The training set vector with $N_{t}U$ features is given by

(6) $$[Z_{1,1},\cdots ,Z_{1,N_{t}},\cdots ,\underset{\mathrm{the}\;i\mathrm{th}\;\mathrm{row}\;\mathrm{of}\;Z}{\underset{︸}{Z_{i,1},\cdots ,Z_{i,N_{t}}}},\cdots ,Z_{U,1},\cdots ,Z_{U,N_{t}}].$$

By repeating the channel generation and data processing *D* times, we obtain a training set matrix $T_{\mathrm{raw}}\in R^{D\times N_{t}U}$ whose rows are given by the training vector in Equation (6). As a special case, when U=1 (single-user communication systems), we use the channel gain as the metric (i.e., $Z_{i,j}=g_{i,j}$) due to the absence of interferences. Since Z is a vector for U=1, we can skip the aforementioned stacking step by directly using Z as the training set vector.

The final step of training set design is to normalize the training samples to obtain a proper input training set for the learning system. Let $T\in R^{D\times N_{t}U}$ denote a training set matrix as one of the inputs to the learning system, whose (i,j)th element, denoted by $T_{i,j}$, is a normalized value of the (i,j)th element of $T_{\mathrm{raw}}$.

(7) $$T_{i,j}=\frac{Z_{i,j}-E_{i}\left(Z_{i,j}\right)}{\max_{i}\left(Z_{i,j}\right)-\min_{i}\left(Z_{i,j}\right)},$$

where the term $\max_{i}\left(Z_{i,j}\right)-\min_{i}\left(Z_{i,j}\right)$ in (7) indicates the normalization used for improving the learning speed/convergence or avoiding a precision issue with very large- or small-value data.

#### 3.1.3. Subtask 1-3. Class Design and Labeling

From the interpretation of the antenna allocation process and multiclass classification, it is clear that designing the labeling is equivalent to designing the antenna allocation scheme. As shown in Section 2.2, the mapping from antenna allocation $s_{l}$ to the index *l* is a one-to-one mapping. Thus, we can use the index set L for the labeling in the machine learning system. Let $c={\left[c_{1},\cdots ,c_{D}\right]}^{T}$ denote the class label vector for the training data matrix T, where $c_{i}\in L$ and i∈{1,⋯,D}. Thus, we use this metric in the following content. The labeling procedure is summarized as follows:

- Evaluate the target KPI, η, for the *d*th channel gain sample with a particular antenna allocation $s_{l}$ corresponding to label l∈L.
- Assign the *d*th element of c, $c_{d}$, with $l^{*}$, which stands for the best choice among all the antenna allocation schemes.
- Repeat the previous two steps for *D* times to go through all *D* training set.

Remark on the reduction of the number of classes: We can further improve the labeling by exploiting the knowledge of a wireless communication system. It is known that multiple antennas with less spatial correlation results in better communication performance, which is also confirmed by our numerical experiments. Therefore, we reduce the number of classes to $L^{\prime }<L$ by deleting some less selected classes, which correspond to the schemes with highly correlated antennas. This elimination can reduce prediction complexity, with a tradeoff in classification performance. Note that even with classes that are uniformly selected, a designer can still reduce *L* to $L^{\prime }$ to reduce the complexity of the learning system if the resultant performance degradation is marginal. On the other hand, the number of clusters can also be automatically determined for unsupervised clustering systems by using Davies-Bouldin or Dunn indices (refer to [55] and references therein).

### 3.2. Task 2: Designing Learning Systems

From Task 1, we obtain the real-value matrix $T\in R^{D\times N_{t}U}$ as the training set and the corresponding class label vector $c={\left[c_{1},\cdots ,c_{D}\right]}^{T}$. Using the labeled training dataset (training data), i.e., T and c, we build a learning system, and specifically, a trained multiclass classifier whose input is an estimated channel gain vector and whose output is the index of the antenna allocation scheme. Since L>2 in our antenna allocation system, we employ *L*-class classification algorithms, such as the multiclass *k*-NN and SVM algorithms. For the simple description of the multiclass classification algorithms, we denote the *i*th row vector of T by $t_{i}\in R^{1\times N_{t}U}$.

We now introduce the fundamental mechanism of a binary SVM classifier and then explain how to perform multiclass classification based on the binary SVM classifier. With a binary SVM, the data are separated into two half-spaces with a hyperplane f(t), which is given by

(8) $$f\left(t\right)=wt^{T}+\beta =0,$$

where $t\in R^{1\times N_{t}U}$ is a feature vector, $w\in R^{1\times N_{t}U}$ is a weight vector, and β∈R is a biasing variable. Here, the linear kernel function, denoted by $K\left(t_{i},t_{j}\right)=t_{i}^{T}t_{j}$, is employed, but generally, various types of kernel functions can also be adopted. This will be discussed later in this section. A classification rule induced by f(t) for the new observation $t_{\mathrm{new}}$ is

(9) $$\mathrm{sign}\left(wt_{\mathrm{new}}^{T}+\beta \right).$$

The training data samples that are nearest to the decision boundary are called support vectors, where the distance is given by $\frac{1}{{∥w∥}_{2}}$. Thus, in order to separate the data as much as possible, the margin that is given by $\frac{2}{{∥w∥}_{2}}$ needs to be maximized. This optimization problem is equivalent to minimizing ${∥w∥}_{2}$. Since the training data may be not totally separable (that is the case for our antenna allocation system), the optimization problem is formulated by introducing slack variable $\xi_{i}\geq 0,\forall i=\left\{1,2,\cdots ,N_{t}U\right\}$ as

(10) $$\begin{array}{cc} \underset{w,\beta }{\mathrm{minimize}} & \frac{1}{2}{∥w∥}_{2}^{2}+C\sum_{i=1}^{N_{t}U}\xi_{i}\\ \mathrm{subject}\;\mathrm{to} & \xi_{i}\geq 0,c_{i}\left(wt_{i}^{T}+\beta \right)\geq 1-\xi_{i}\;\forall i, \end{array}$$

where the “penalty” parameter *C* is used to penalize the training error of the soft margin SVM. This parameter *C* needs to be tuned for good classification performance because too large a *C* causes overfitting and too small a *C* causes underfitting. The decision boundary can be found by solving this convex quadric optimization problem. As mentioned before, by adopting different kernel functions, we can apply the “kernel trick” to map the original feature space to a higher-dimensional feature space where the training set could be more separable. For instance, the polynomial kernel function and the Gaussian kernel function are given by

(11) $$K\left(t_{i},t_{j}\right)={\left(1+t_{i}^{T}t_{j}\right)}^{p}$$

and

(12) $$K\left(t_{i},t_{j}\right)=e^{\frac{-∥t_{i}-t_{j}{∥}^{2}}{2\sigma^{2}}},$$

respectively, where p≥2 is the polynomial power and $\sigma^{2}$ is the variance. We can do this because the optimization process of SVM allows us to simply modify the kernel function $K(t_{i},t_{j})$ by replacing the linear kernel function with other kernel functions, without changing the overall optimization algorithm.

In order to perform multiclassification using the binary SVM method that was originally designed for binary classification [56], we can either employ the one-vs-all (OVA) strategy with *L* binary SVM learners or the one-vs-one (OVO) strategy with $\frac{L(L-1)}{2}$ binary SVM learners [57]. Compared to the OVO strategy, the OVA strategy has a lower computational complexity because fewer binary SVM learners are required. The selection of the proper multiclassification strategy and kernel function will be discussed in detail based on numerical results in the next subsection.

### 3.3. Task 3. Parameter Adjustment

**Figure 3:**
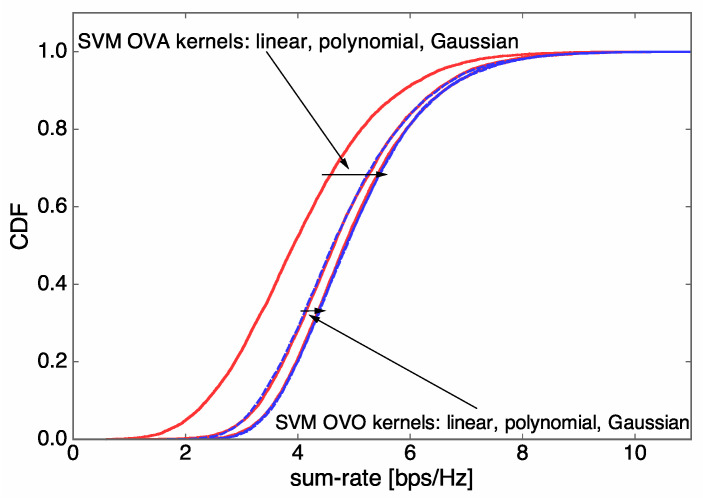
Support vector machine (SVM) performance evaluation results when $N_{t}=5$ and U=3: the empirical cumulative density function (CDF) of the sum rate for various SVM kernel functions.

**Figure 4:**
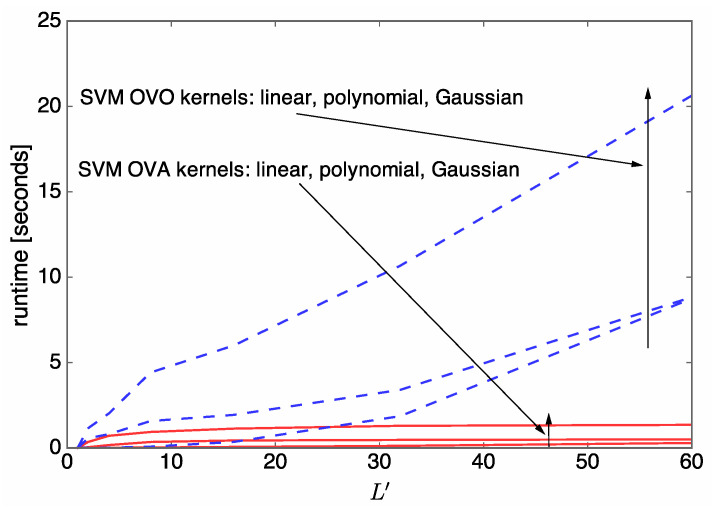
SVM performance evaluation results when $N_{t}=5$ and U=3: runtime over $L^{\prime }$ for various SVM kernel functions.

For the multiclass SVM algorithms, several parameters and strategies can be selected and tuned in order to achieve superior sum-rate performance. Here, we note that machine learning algorithms with low prediction complexity are favorable to antenna selection in communication systems. Based on numerical results regarding the sum-rate performance and prediction runtime performance, we discuss the selection of kernel function from linear, polynomial, and Gaussian kernel functions, and the selection of multiclass strategy from between OVA and OVO. Through rigorous simulation and comparison, we find that the SVM algorithm with the OVA strategy and the Gaussian kernel provides nearly the best sum-rate performance with a relatively low prediction runtime, and is thus adopted in our antenna selection system. For example, Figure 3 and Figure 4 verify our observation when $N_{t}=5$ and U=3, where solid red curves represent the use of SVM algorithms with the OVA strategy while dashed blue curves represent the use of SVM algorithms with the OVO strategy. In contrast, SVM algorithms with the OVO algorithm show a relatively high prediction runtime that grows fast with $L^{\prime }$, although the sum-rate performance is slightly superior to that of the OVA counterparts. The SVM algorithm with the OVA strategy and linear kernel provides the lowest prediction runtime performance but the sum-rate performance degradation is severe.

For each combination of multiclass strategies and kernel functions, proper values of crucial parameters such as the variance $\sigma^{2}$ and the penalty parameter *C* are found by extensive experimentation via a heuristic search targeting the minimum cross-validation loss. For instance, when the OVA strategy and the Gaussian kernel function are adopted, $\sigma^{2}$ and *C* are determined by 21.56 and 7.67, respectively.

## 4. Numerical Evaluation

In this section, we evaluate the performance of the designed communication system invoking SVM-based allocation with the OVA strategy and the Gaussian kernel function in terms of sum rate and computational complexity via computer simulations. For comparison, we also consider three benchmark systems: (i) OPT, which is an optimization-driven method that maximizes the sum rate, i.e., exhaustive search, discussed in Section 2.3, (ii) RAND, which selects antennas randomly, (iii) and *k*-NN, an antenna allocation system based on the *k*-NN algorithm instead of SVM. Unless otherwise stated, we take into account full permutations of an antenna set (i.e., full classes), e.g., L=60 when $N_{t}=5$ and U=3. We also evaluate the performance with $L^{\prime }<L$, that is, the reduced number of classes for computational efficiency. In our simulations, the number of training samples is set by $4.9\times 10^{4}$, i.e., $D=4.9\times 10^{4}$, which is the number of rows in the training data matrix T. For a *k*-NN algorithm, we set $k=\frac{D}{100}$ and use a Euclidian distance metric for the best classification accuracy of the antenna allocation system. For ease of presentation, we evaluate the performance by limiting the number of users, *U*, to certain values (e.g., U=3). However, we can adopt any *U* in our system by generating multiple machine learning models offline according to various *U*s and then choosing one trained model for a given *U*.

### 4.1. Sum-Rate Performance

**Figure 5:**
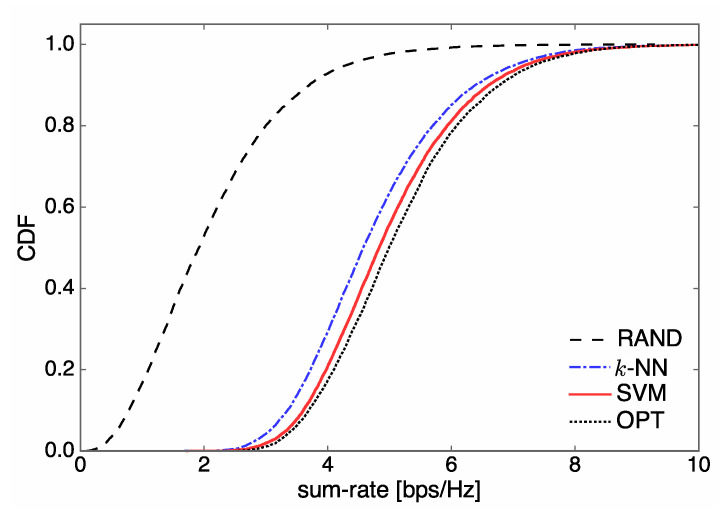
Empirical CDF of sum rate when $N_{t}=5$ and U=3.

The sum-rate performance of SVM designed in the previous section is compared to the other schemes. In Figure 5, we illustrate the cumulative density function (CDF) of the sum rate when $N_{t}=5$ and U=3. It can be seen that the SVM classifier provides the closest sum-rate performance to that of OPT. The *k*-NN classifier achieves superior sum-rate performance to that of RAND, yet it is highly inferior to the proposed SVM.

**Figure 6:**
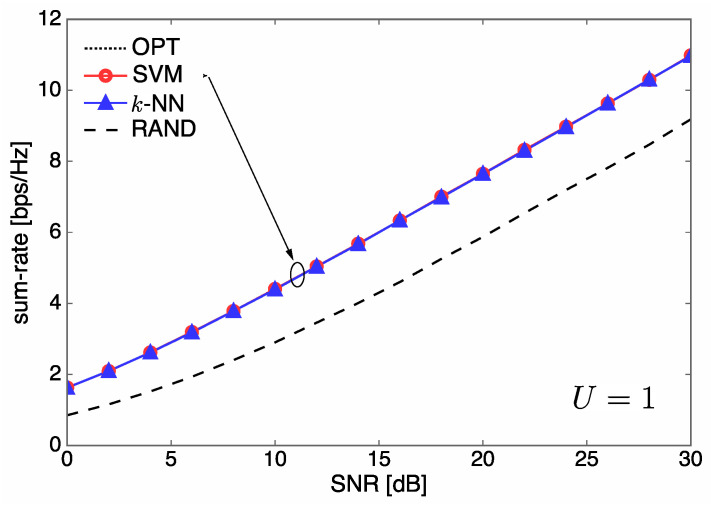
Sum rate over signal-to-noise ratio (SNR) performance for different system configurations when $N_{t}=5$ and U=1.

**Figure 7:**
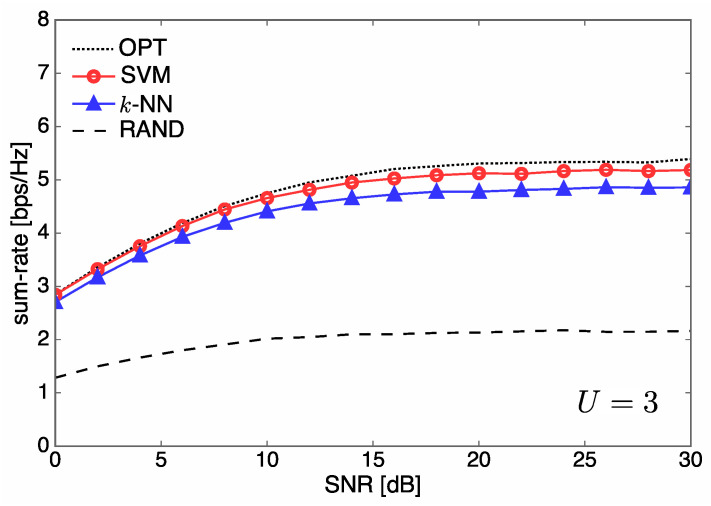
Sum rate over SNR performance for different system configurations when $N_{t}=5$ and U=3.

In Figure 6 and Figure 7, we illustrate the sum rate over SNR for different numbers of users U∈{1,3}, when $N_{t}=5$. For U=1 corresponding to single-user communications with no interference, the sum rate curves of SVM and *k*-NN almost coincide with that of OPT due to the excellent classification accuracy of our learning system. On the other hand, for U=3, we observe that SVM outperforms *k*-NN, as shown in Figure 5. The sum-rate performance of all methods gets saturated in a high SNR regime because our communication system is interference-limited.

**Figure 8:**
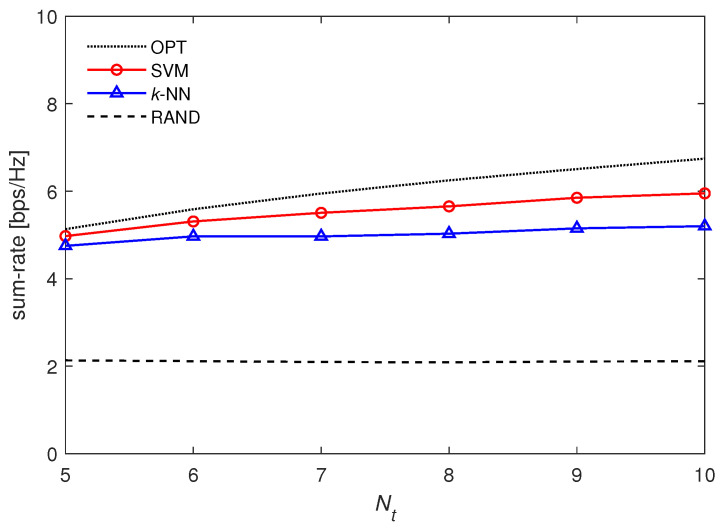
Sum rate over $N_{t}$ when U=3.

In Figure 8, we plot the sum rate over $N_{t}$ from 5 to 10 when U=3. The number of classes *L* is set according to the value of $N_{t}$, e.g., L=10!/7!=720 for $N_{t}=10$. We observe that the sum rate of SVM and OPT increases with $N_{t}$ with the help of the multi-antenna selection diversity gain. Interestingly, the sum-rate performance of *k*-NN tends to saturate with increasing $N_{t}$ due to the limited number of training samples. More specifically, while there are 720 classes, the number of training samples is not sufficient to guarantee classification performance in the nearest neighbor search. Thus, SVM is shown to be more robust to various system configurations scalable with $N_{t}$.

**Figure 9:**
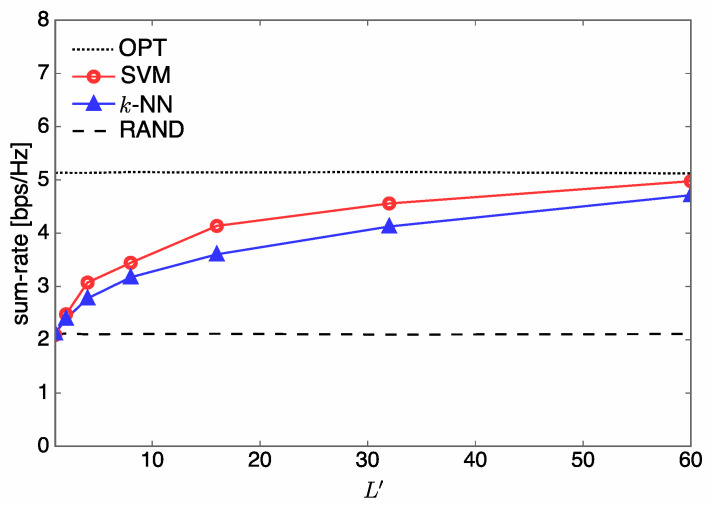
Sum rate over $L^{\prime }$ when $N_{t}=5$ and U=3.

In Figure 9, we compare the sum rate over the number of classes $L^{\prime }$ when $N_{t}=5$, U=3 and $L^{\prime }\in \left\{1,2,4,8,16,32,60\right\}$. It is observed that the sum rates of SVM and *k*-NN coincide with that of RAND when $L^{\prime }=1$, and they increase monotonically with $L^{\prime }$.

**Figure 10:**
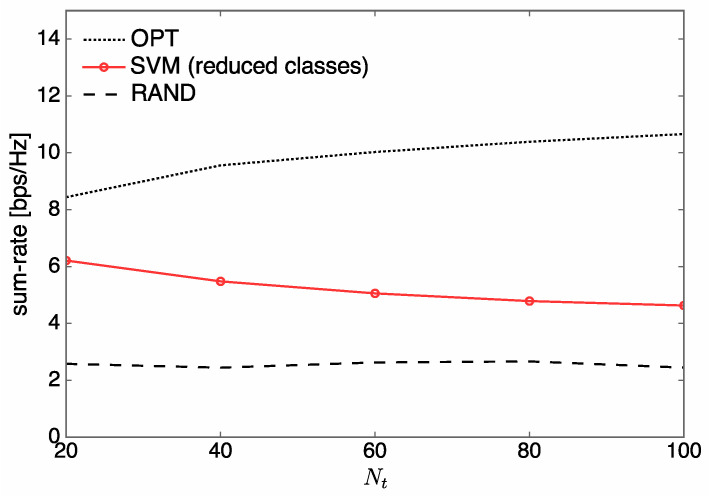
Sum rate over $N_{t}$ when $L^{\prime }=60$ and U=2.

To demonstrate our SVM-based approach in a massive multi-antenna setting, in Figure 10 we plot the sum rate over $N_{t}$ from 20 to 100 when U=2 and $L^{\prime }=60$. It is observed that, unlike the results in Figure 10, the sum rate of SVM is reduced with increasing $N_{t}$. Such a degradation occurs because the number of classes, $L^{\prime }$, is over-reduced for large $N_{t}$. To overcome this problem, $L^{\prime }$ needs to scale according to the size of $N_{t}$, which is not employed in our study, however, since such a scaling of $L^{\prime }$ should be accompanied by a much larger training dataset, which may cause a memory overflow, in order to guarantee a sum-rate performance comparable to that of OPT. Using a sophisticated offline training method to appropriately adjust the number of classes without any memory overflow remains a goal for future work. However, it can be seen that SVM still offers substantial gains in terms of sum rate compared to RAND.

### 4.2. Complexity Analysis and Runtime Evaluation

Selection complexities are compared in Table 2. The complexity of the optimization-based algorithm is discussed in Section 2.2, and the complexity of random antenna selection is O(1) because the only task is to generate a random integer. For the machine learning-based algorithms, selection complexity is defined as the online prediction complexity, excluding the training complexity since training can be completed offline with more powerful computing resources before the communication phase. In the *k*-NN algorithm, the complexity of $O\left(DN_{t}U\right)$ and $O\left(Dk\right)$ comes from the computing distances to all training samples and finding the *k* nearest neighbors, respectively [58]. In multiclass SVM algorithms, for the OVO strategy, because $M=\frac{L(L-1)}{2}$ binary SVM learners are employed for the multiclass prediction, complexity is given by $O\left({L^{\prime }}^{2}N_{t}U\right)$; while for the OVA strategy, *L* binary SVM learners are required, and thus complexity is $O\left(L^{\prime }N_{t}U\right)$ [57]. Note that the complexity of the learning-based methods mainly depends on the number of classes, and the number of classes can be reduced from *L* to $L^{\prime }$, as discussed in Section 3.1. From Table 2, it can be seen that the selection complexity of the machine learning-based algorithms (*k*-NN and SVM) is polynomial on $N_{t}$ and *U*, which is lower than that of the optimization-based algorithm using exhaustive search among all potential antenna allocation schemes.

**Figure 11:**
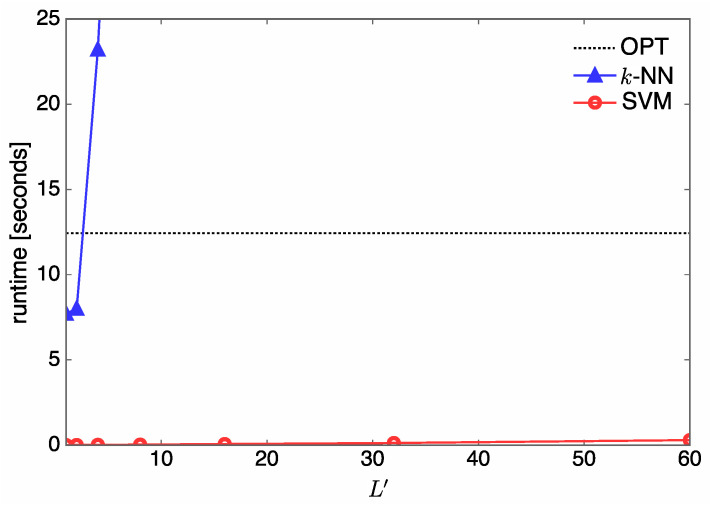
Runtime over $L^{\prime }$ when $N_{t}=5$ and U=3.

Now, the runtime complexity (in seconds) of the online antenna selection is evaluated under the various simulation environments. Figure 11 shows runtime complexity over $L^{\prime }$ when $N_{t}=5$, U=3, and $L^{\prime }\in \left\{1,2,4,8,16,32,60\right\}$. It can be seen that the complexity of *k*-NN is much higher than that of OPT when $L^{\prime }\geq 4$. Since the complexity of *k*-NN is proportional to *D*, as shown in Table 2, the complexity of *k*-NN can be reduced at the cost of a degraded sum rate. On the other hand, the complexity of SVM is much lower than that of OPT; therefore, SVM is favorable for our antenna selection system.

**Figure 12:**
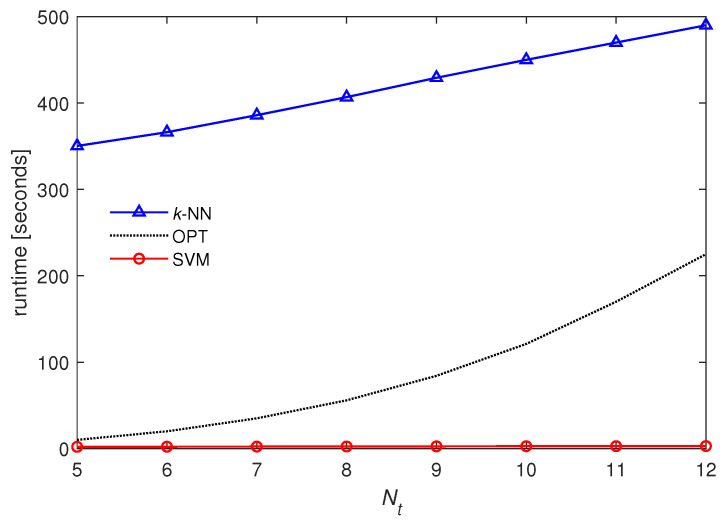
Runtime over $N_{t}$ when U=3 and $L^{\prime }=32$.

In Figure 12, we plot the runtime complexity over $N_{t}$ from 5 to 12 when U=3. We observe that the complexity of SVM is further reduced and remarkably lower than that of OPT when $L^{\prime }=32$. More specifically, the complexity of SVM tends to increase slowly on a linear scale with $N_{t}$, whereas that of OPT dramatically increases as $N_{t}$ increases (refer to Table 2). However, the complexity of *k*-NN is still much greater than that of OPT.

## 5. Concluding Remarks and Future Work

In this paper, we introduced a new framework for applying multiclass classification to an antenna allocation system with multiple antennas in multiuser downlink communications, under the assumption of channel amplitude information at the transmitter. The proposed antenna allocation system based on an SVM multiclass classifier was numerically evaluated and verified based on sum-rate performance and computational complexity. The following main results were obtained: (i) If the number of classes $L^{\prime }$ is suitably established, then the sum-rate performance of SVM is comparable to that of the optimization-driven method and significantly reduces computational complexity in the online antenna section; (ii) the classification performance of *k*-NN is inferior but still comparable to that of SVM; and (iii) for a given $L^{\prime }$, the runtime complexity of the SVM classifier increases linearly with the number of antennas, which implies that the designed learning-based approach using SVM is appropriate, especially for large-scale antenna systems. Suggestions for future research in this area include (i) developing a variety of learning systems by precisely designing training data along with more channel information (e.g., channel phase information) in interference-limited multiuser communications, (ii) supporting multi-antenna users, and iii) developing an online learning algorithm to track channels with time-varying statistics.

## Figures and Tables

**Table 1:** Notations used to describe communication systems.

Notation	Description
$N_{t}$	number of antennas at the transmitter
*U*	number of users
$h_{i}$	channel coefficient vector to device *i*
H	overall channel coefficient matrix
G	overall channel gain matrix
$s_{l}$	index vector of the allocated antenna with label *l*
L	set of labels for all the available antennas allocated
*L*	number of labels in L
$P_{\mathrm{TX}}$	transmit power per antenna
$R_{\mathrm{sum}}$	sum rate of the system in bps/Hz

**Table 2:** Performance and allocation complexity of the algorithms. OVO: one-vs-one strategy; OVA: one-vs-all strategy

Algorithm	OPT	RAND	*k*-NN	SVM
Sum-rate performance	Best	Worst	Close to SVM	Second-best
Allocation complexity	$O\left(\frac{U^{2}N_{t}!}{(N_{t}-U)!}\right)$	O(1)	$O\left(DN_{t}U+Dk\right)$	$O\left({L^{\prime }}^{2}N_{t}U\right)$ for OVO and $O\left(L^{\prime }N_{t}U\right)$ for OVA
